# Editorial: Stroke research in the elderly: addressing ageism and prognostication

**DOI:** 10.3389/fstro.2026.1978900

**Published:** 2026-09-22

**Authors:** Hipólito Nzwalo, Miguel Barboza, Nicola Logallo

**Affiliations:** 1 Faculty of Medicine and Biomedical Sciences, University of Algarve, Faro, Portugal; 2 Algarve Biomedical Center Research Institute, Faro, Portugal; 3 Hospital Rafael Angel Calderon Guardia, San José, Costa Rica; 4 Department of Neurosurgery, Haukeland University Hospital, Bergen, Norway

**Keywords:** ageism, biomarkers, prognostication, stroke, very old adults

Nearly one-third of strokes occur in individuals over 80 years of age (Russo et al., 2011), yet despite the increasing representation of older and very old patients within stroke populations, evidence to guide their management and prognostication remains scarce. Age-related gaps in the use of evidence-based prevention and treatment persist despite overall improvements in stroke care over time (Fonarow et al., 2010).

Consequently, clinicians often extrapolate findings from studies conducted in younger and healthier cohorts to a group whose physiological reserve, comorbidity burden, and goals of care are fundamentally different. For instance, when applied to very old patients, the main mortality scores for intracerebral hemorrhage retain acceptable overall discriminative performance but lose accuracy in identifying specific subgroups at higher risk of death (Batista et al., 2023). This ageism is reflected in the care of older stroke patients, with evidence showing that older survivors receive less guideline-compliant care than younger patients, regardless of functional status on admission or discharge (Young, 2006).

This Research Topic presents a broad range of studies addressing key aspects of stroke in the very old, encompassing risk prediction, prognostication, acute management, and long-term outcomes.

Several articles focused on risk stratification for incident stroke in the elderly using readily available data. These include the Weight-Adjusted Waist Index, an innovative obesity metric (Li et al.), the combined levels of C-reactive protein and LDL cholesterol (Lv et al.), and the glucose disposal rate, a surrogate marker of insulin resistance (Zhao et al.).

Two studies examined intermediate risk factors for stroke, such as arteriosclerosis (Gao, Zhang et al.) and cerebral white matter hyperintensity (WMH) (Ji et al.). Obesity, hypertension, triglycerides, glycosylated hemoglobin, sex, and fasting blood glucose were identified as predictors of arteriosclerosis (Gao, Zhang et al.) and higher plasma levels of trimethylamine N-oxide, a metabolite of gut microbiota, were associated with greater WMH burden (Ji et al.). Importantly, the presence of WMH was associated with early progression of stroke particularly in older patients with anterior choroidal artery infarction (Gao, Wang et al.).

Psychological factors were likewise shown to influence stroke risk. Ascending trajectory of cognitive-affective symptoms, alongside unstable and persistently elevated trajectories of somatic depressive symptoms, were associated with increased stroke risk (Zhang et al.).

Several studies highlighted the value of inflammatory and metabolic biomarkers for functional and survival prognostication in older stroke patients. The neutrophil-percentage-to-albumin ratio (Huang et al.), the triglyceride-glucose-body mass index (Zheng J. et al.), and the neutrophil-to-lymphocyte ratio (Zheng J. et al.) emerged as promising predictors of mortality. These findings underscore the growing role of accessible blood-based biomarkers in risk assessment and outcome prediction among elderly stroke survivors.

A comprehensive review synthesized current evidence regarding the role of deubiquitinases, key regulators of the ubiquitin-proteasome system implicated in cerebral ischemia-reperfusion injury. The authors discussed the potential of deubiquitinase-targeted interventions as novel neuroprotective strategies (Qin et al.).

In the setting of acute reperfusion therapies, several biomarkers demonstrated prognostic utility: a risk model integrating cerebral circulation time and collateral status improved the prediction of futile recanalization following mechanical thrombectomy (Song et al.).

Distinct trajectories of the neutrophil-to-albumin ratio were associated with functional outcomes after endovascular treatment (Gao, Sun et al.) while the triglyceride-glucose-body mass index predicted long-term all-cause mortality in elderly patients treated with intravenous thrombolysis (Cheng et al.). In addition, the platelet-to-lymphocyte ratio emerged as an independent predictor of mortality in patients with severe acute ischemic stroke requiring intensive care (Liu G. et al.).

Some contributions challenged ageist assumptions in stroke care by demonstrating that evidence-based interventions remain beneficial in elderly patients. Studies showed extracranial vertebral artery stenting (Wu and Zhang) and intravenous thrombolysis for minor ischemic stroke (Naess) can be both safe and effective in older individuals, supporting treatment decisions based on biological rather than chronological age.

Long-term cognitive outcomes were also addressed. Chen et al. identified three distinct post-stroke cognitive trajectories among elderly patients: “high cognitive function–slow decline,” “low cognitive function–intermediate decline,” and “low cognitive function–rapid decline.” Furthermore, the hemoglobin-to-red cell distribution width ratio was associated with the risk of post-stroke cognitive impairment, suggesting a readily available biomarker for identifying patients at greater risk of cognitive deterioration (Liu Y. et al.).

Across most contributions, the discussion of results confirmed that the evidence for very old stroke patients remains weak, fragmented, and incomplete, reinforcing that tools and guidelines derived from younger, less comorbid populations cannot simply be extrapolated to this group (Fonarow et al., 2010). By addressing this diversity of topics, the studies included in this Research Topic make an important contribution to the understanding of stroke in older and very old adults. They advance the development of innovative strategies for risk prediction, prognostication, and personalized management, while simultaneously helping to address persistent age-related disparities in stroke research and clinical care.

## References

[B1] Batista R. Pereira M. Catamo Vaz D. Buque H. Nzwalo H. Marreiros A. . (2023). Prognostic accuracy of common mortality prognostic scales in very old patients with intracerebral haemorrhage. Ann. Neurosci. 32, 161–166. doi: 10.1177/09727531231185200 PMC12267898 40688404

[B2] Fonarow G. C. Reeves M. J. Zhao X. Olson D. M. Smith E. E. Saver J. L. . (2010). Get with the guidelines-stroke steering committee and investigators. Age-related differences in characteristics, performance measures, treatment trends, and outcomes in patients with ischemic stroke. Circulation 121, 879–891. doi: 10.1161/CIRCULATIONAHA.109.892497 20142445

[B3] Russo T. Felzani G. Marini C. (2011). Stroke in the very old: a systematic review of studies on incidence, outcome, and resource use. J. Aging Res. 2011:108785. doi: 10.4061/2011/108785 21876804 PMC3161203

[B4] Young J. (2006). Ageism in services for transient ischaemic attack and stroke: could be cut by emulating successful efforts against ageism in heart disease care. BMJ 333, 508–509. doi: 10.1136/bmj.38961.641400.BE 16960188 PMC1562462

